# Expression analysis of *DIO2*, *EYA3*, *KISS1* and *GPR54* genes in year-round estrous and seasonally estrous rams

**DOI:** 10.5194/aab-63-451-2020

**Published:** 2020-12-09

**Authors:** Qing Xia, Ran Di, Xiao-Yun He, Cai-Hong Wei, Ming-Xing Chu

**Affiliations:** Key Laboratory of Animal Genetics and Breeding and Reproduction of the Ministry of Agriculture and Rural Affairs, Institute of Animal Science, Chinese Academy of Agricultural Sciences, Beijing 100193, PR China

## Abstract

The expression characteristics of the hypothalamic–pituitary–gonadal (HPG) axis-related candidate genes, *DIO2*, *EYA3*, *KISS1* and *GPR54*, were analyzed in year-round estrous rams (small-tail Han sheep, STH) and seasonally estrous rams (Sunite sheep, SNT) using qPCR. The results were as follows: *DIO2* was mainly expressed in pituitary, and *KISS1* was specifically expressed in hypothalamus in the two groups. However, *EYA3* and *GPR54* were widely expressed in the cerebrum, cerebellum, hypothalamus, pituitary, testis, epididymis, vas deferens and adrenal gland tissues in both breeds, with significant differences in the cerebellum, hypothalamus, pituitary, testis and vas deferens tissues. We speculated that *DIO2* and *KISS1* may have positive roles in different regions in ram year-round estrus. Moreover, the expression patterns of* EYA3* and *GPR54 *suggested that they may regulate the estrous mode of ram via testis and vas deferens. This is the first study to systematically analyze the expression patterns of HPG axis-related genes in rams.

## 1 Background

Seasonal estrus is an important physiological behavior that animals have evolved to adapt to the environment, which is regulated by photoperiods (Dupré et al., 2010; Dardente et al., 2019). The photoperiodic mechanism in vertebrates is known to involve seasonal regulation of thyroid hormones, mediated in mammals via specialized cells (thyrotrophs) in the pars tuberalis (PT). Normally, the female estrous cycle is initiated by the melatonin from the hypothalamus, which functions in the pituitary and regulates the expression of photoperiodically induced genes (eyes absent 3 (*EYA3*), thyrotrophin β subunit (*TSH*β) and chromogranin (*CGA*)). Then it induces a neuroendocrine cascade along the hypothalamic–pituitary–gonadal (HPG) axis in turn (Dupré et al., 2010; Tavolaro et al., 2014).

To date, some major genes affecting seasonal estrus in sheep have already been identified, such as period (*PER*), cryptochrome (*CRY*), *BMAL1* and *CLOCK*, which all belong to the circadian rhythm genes. The expression levels of these genes are closely related to cycles in behavior and physiology (Marcheva et al., 2010; Janich et al., 2011; Nam et al., 2015).

Research has also found that HPG axis-related genes, type 2 deiodinase (*DIO2*), eyes absent 3 (*EYA3*), G protein-coupled receptor 54 (*GPR54*) and kisspeptin-1 (*KISS1*), are involved in regulating the mechanism of seasonal estrus in mammals (Dupré et al., 2010; Herbison et al., 2010; Tariq et al., 2013; Liu et al., 2017). The variations of *DIO2/3 *expression patterns in hypothalamic ependymal cells are regulated by melatonin secretion, which is associated with inactivation of THs and related to gonadal function (Lincoln, 1999; Ikegami and Yoshimura, 2012). *EYA3*, as the strongest long photoperiod-induced gene in sheep, is activated by a long photoperiod, revealing a common photoperiodic molecular response in birds and mammals (Dupré et al., 2010). *GPR54* has been identified as the receptor of *KISS1* (Muir et al., 2001; Ohtaki et al., 2001); it is vital for puberty onset and male fertility due to its central regulatory roles (Han et al., 2020). The loss-of-function mutations in *GPR54 *for humans and mice lead to the symptoms of idiopathic hypogonadotropic hypogonadism with the phenomenon of retarded sexual development and failure to reach puberty (Funes et al., 2003; Seminara et al., 2003; Roux et al., 2003). *KISS1* neurons in the arcuate nucleus may regulate the negative feedback effect of gonadal steroids on GnRH and gonadotropin secretion in both sexes (Popa et al., 2008). Kisspeptins are potent secretagogues for GnRH, and the *KISS1* gene is a target for regulation by gonadal steroids (e.g., estradiol and testosterone), metabolic factors (e.g., leptin), photoperiods, and seasons (Li et al., 2008). With an understanding of the associations of the four genes with seasonal estrus in female animals, there was a desire to gain further knowledge of how these genes could affect male reproduction.

Sheep (*Ovis aries*) are a typical seasonally estrous species (Tang et al., 2018). Small-tail Han sheep (STH) and Sunite sheep (SNT) are two native Chinese sheep breeds. In addition, STH has comparatively excellent characters, such as crude feed tolerance, rapid growth and good meat quality. Therefore, STH sheep provide an ideal model breed to explore the molecular genetic mechanisms related to year-round estrus in certain breeds (Miao et al., 2016). In contrast, Sunite sheep develop gonads and display seasonal reproductive behavior during specific times of the year (La et al., 2020). Therefore, the molecular mechanism of seasonal reproduction of sheep can be better studied by using Sunite sheep as a model. Results of all these previous studies indicated the *DIO2*, *EYA3*, *KISS1* and *GPR54* genes have important functions in female estrus. However, there have been few studies on the expression analyses in rams. Whether the four genes affect rams estrus remains to be elucidated. Therefore, in this study, we compared the tissue expression profiles and mRNA expression levels of the four genes in eight reproduction-related tissues between STH and SNT rams. Our study paves the way for an in-depth study of the seasonally estrous mode of rams.

## 2 Materials and methods

### 2.1 Ethics statement and sample collection

All animals used in this study were approved by the Science Research Department (in charge of animal welfare issues) of the Institute of Animal Science, Chinese Academy of Agricultural Sciences (IAS-CAAS; Beijing, PR China). In addition, there was ethics approval by the animal ethics committee of IAS-CAAS (no. IAS2020-82, 28 July 2020).

Three STH rams were supplied by the Yuncheng Breeding Sheep Farm (Shandong province, China), and three SNT rams were from Urad Middle Banner, Inner Mongolia of China. All rams were healthy, approximately 2.5 years old and were kept in a sheltered outdoor paddock. All animals were euthanized (intravenous pentobarbital at 100 mg per kilogram), and eight tissues (cerebrum, cerebellum, hypothalamus, pituitary, epididymis, testis, vas deferens and adrenal gland) were collected from each animal. All tissues were frozen in liquid nitrogen immediately and then stored at -80 ${}^{\circ }$C to be used for RNA extraction.

### 2.2 Total RNA extraction and cDNA synthesis

Total RNA was extracted from the eight collected tissues using a total RNA extraction kit for animal tissue (Tiangen, Beijing, China). Trizol (Invitrogen Inc., Carlsbad, CA, USA) was used to dissolve the tissues. The quantity and quality of total RNA were monitored using 1.5 % agarose gel electrophoresis (U = 150 V; 10 min) and ultraviolet spectrophotometry (UV-1201, Shimadzu, Kyoto, Japan), respectively. Then, the RNAs were stored at -80 ${}^{\circ }$C until use.

**Table 1:** Primers of studied genes.

Gene name	Primer sequence ($5^{\prime }$-$3^{\prime }$)	Length (bp)	$T_{m}$ (${}^{\circ }$C)	Accession no.
*DIO2*	F: GAAGGAATGCGCTGCATCTG R: GGGAATTGGGGGCATCTTCA	82	60	XM_027972090.1
*EYA3*	F: GGATCCTATGCCCAGAAGTATG R: CCACATCTTCCACATGCACC	106	61	NM_001161733.1
*GPR54*	F: TCGGACAAAAGCGAGACCCAGGAGGC R: TGAAGGGCACGCAGCACAGCACCAA	146	60	NM_001318077.1
*KISS1*	F: TCATTTGGAGACCCCTCAGGA R: ATCCTGGTGAGAAGATGCCG	70	60	NM_001306104.1
*GAPDH*	F: GGTGATGCTGGTGCTGAG R: TGACAATCTTGAGGGTGTTG	181	60	NM_001190390.1

The first strand of cDNA was prepared using a PrimeScript™ RT Reagent Kit according to the manufacturer's instructions (TaKaRa Bio Inc., Dalian, China). The PCR thermocycler program was as follows: 37 ${}^{\circ }$C for 15 min, followed by 85 ${}^{\circ }$C for 5 s. The reaction mixture contained 1.0 µL of PrimeScript RT Enzyme, 1.0 µL of random 6-mers, 4.0 µL of 5 × PrimeScript Buffer (for real time), 1.0 µL of total RNA and 13 µL of RNase-free ddH${}_{2}$O (total volume, 20 µL). Prior to storage at -80 ${}^{\circ }$C, the standard working concentration of cDNA was 200 ng/µL. The quality of cDNA was evaluated by housekeeping gene (*GAPDH*) amplification, and cDNA was stored at -20 ${}^{\circ }$C until use.

### 2.3 Primer design

Primers were designed with Premier 3.0 (version 4.1.0) (https://bioinfo.ut.ee/primer3-0.4.0/, last access: 15 October 2019). A total of five pairs of primers were designed to amplify different fragments of the ovine *DIO2* (GenBank: XM_027972090.1), *EYA3* (GenBank: NM_001161733.1), *GPR54* (GenBank: NM_001318077.1), *KISS1* (GenBank: NM_001306104.1) and *GAPDH* (GenBank: NM_001190390.1) genes, based on their assembled sequences in GenBank. All primers were synthesized by Beijing Tianyi Biotechnology Co., Ltd. (Beijing, China). The housekeeping gene (*GAPDH*) was used as an internal control to normalize the threshold cycle ($C_{t}$) values. Primer details are given in Table 1.

**Figure 1:**
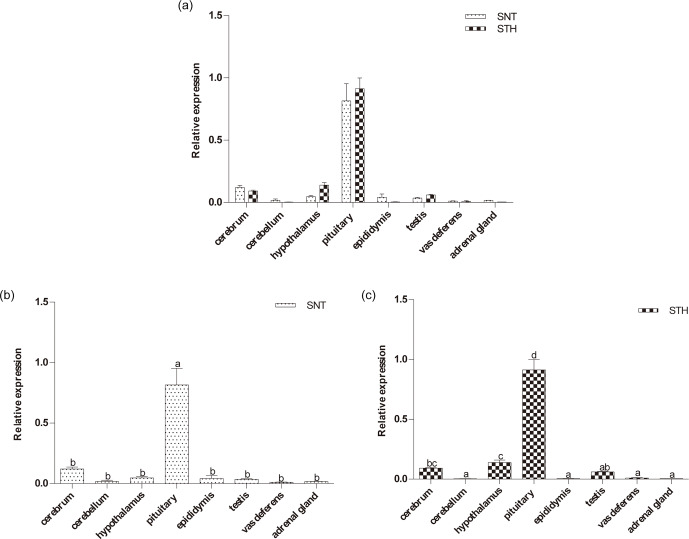
The expression of *DIO2* in eight tissues of small-tail Han sheep (STH) and Sunite sheep (SNT): **(a)** the comparison of the expression between SNT and STH, **(b)** the expression in SNT and **(c)** the expression in STH. Means with different superscripts are significantly different (P<0.05). The significant results with a p value lower than 0.05 are given one asterisk (${}^{*}$) and lower than 0.01 are given two asterisks (${}^{**}$), respectively.

### 2.4 qPCR

Real-time polymerase chain reaction (qPCR) amplification was performed in 20 µL of reaction mixture that contained 10 µL of SYBR Premix EX Taq II (TaKaRa Bio Inc., Dalian, China), 0.4 µL of each forward and reverse primer, 6.4 µL of RNase-Free ddH${}_{2}$O, and 2 µL of cDNA. PCR amplification was performed in triplicate wells using the following conditions: initial denaturation at 95 ${}^{\circ }$C for 5 min, followed by 40 cycles of 95 ${}^{\circ }$C for 10 s and 60 ${}^{\circ }$C for 30 s. The dissociation curve was analyzed after amplification. A melting temperature ($T_{\text{m}}$) peak at 85 ${}^{\circ }$C ± 0.8 on the dissociation curve was used to determine the specificity of PCR amplification.

**Figure 2:**
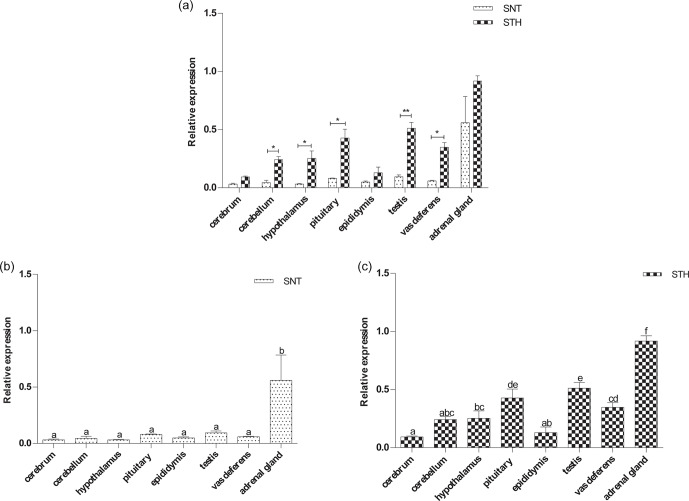
The expression of *EYA3* in eight tissues of small-tail Han sheep (STH) and Sunite sheep (SNT): **(a)** the comparison of the expression between SNT and STH, **(b)** the expression in SNT and **(c)** the expression in STH. Means with different superscripts are significantly different (P<0.05). The significant results with a p value lower than 0.05 are given one asterisk (${}^{*}$) and lower than 0.01 are given two asterisks (${}^{**}$), respectively.

### 2.5 Statistical analysis

The relative gene expression levels were calculated by the 2${}^{-\Delta \Delta C_{t}}$ method (Livak and Schmittgen, 2001; Livak, 2008). Statistical analyses were carried out using SPSS 19.0 software (IBM, Armonk, NY, USA). The levels of gene expression were analyzed for significant differences by one-way analysis of variance (ANOVA) followed by Fisher's least significant difference test as a multiple comparison test (Meier, 2006). All experimental data are presented as mean ± standard error of the mean (SEM).

## 3 Results

### 3.1 Expression level of *DIO2*

As shown in Fig. 1, the expression level of *DIO2* was similar in SNT and STH, with the highest level being in pituitary, followed by hypothalamus and cerebrum, with no significant difference between the two sheep breeds (Fig. 1a). However, *DIO2* expression in pituitary was significantly higher than in the other seven tissues both in SNT and STH (P<0.05), and the expression level in hypothalamus was significantly higher than that in cerebellum, epididymis, testis, vas deferens and adrenal gland in STH (P<0.05) (Fig. 1b, c).

### 3.2 The expression level of *EYA3*

The results of the *EYA3* expression analysis are shown in Fig. 2. *EYA3* was widely expressed in the eight tissues in the two breeds. And *EYA3* was expressed significantly higher in STH than SNT except for cerebrum and adrenal gland (P<0.05, P<0.01) (Fig. 2a). Additionally, *EYA3* expression was significant higher in adrenal gland than that in other tissues both in SNT and STH (P<0.05) (Fig. 2b, c). Its expression in testis was significantly higher than other six tissues, with pituitary significantly higher than those of cerebrum, cerebellum, hypothalamus and epididymis in STH (P<0.05) (Fig. 2c). The expression of *EYA3* in cerebellum, hypothalamus and epididymis had no significant difference in STH (Fig. 2c).

**Figure 3:**
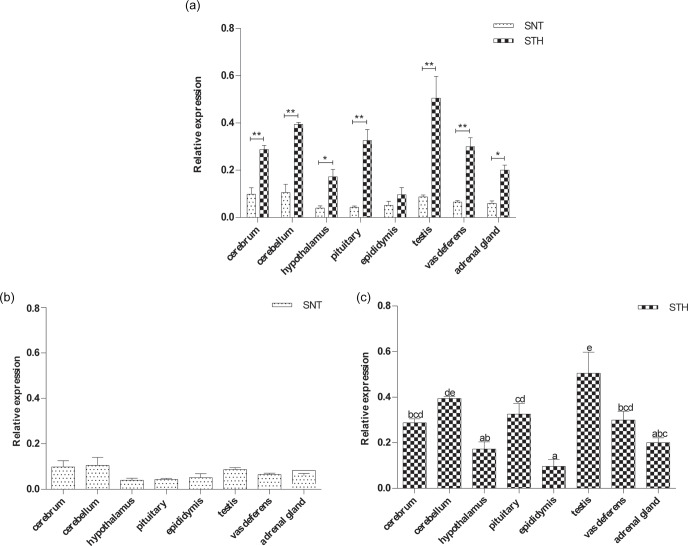
The expression of *GPR54* in eight tissues of small-tail Han sheep (STH) and Sunite sheep (SNT): **(a)** the comparison of the expression between SNT and STH, **(b)** the expression in SNT and **(c)** the expression in STH. Means with different superscripts are significantly different (P<0.05). The significant results with a p value lower than 0.05 are given one asterisk (${}^{*}$) and lower than 0.01 are given two asterisks (${}^{**}$), respectively.

### 3.3 The expression level of *GPR54*

Figure 3 clearly shows that *GPR54* was widely expressed in all tissues in the two breeds, with a higher level in STH than SNT. The expression levels of *GPR54* were reached at significant difference in hypothalamus and adrenal gland (P<0.05), with extremely significant difference in cerebrum, cerebellum, pituitary, testis and vas deferens between the two breeds (P<0.01) (Fig. 3a). There was no significant difference in SNT (Fig. 3b). And it was the highest in testis and the lowest in epididymis in STH (P<0.05) (Fig. 3c).

### 3.4 The expression level of *KISS1*

**Figure 4:**
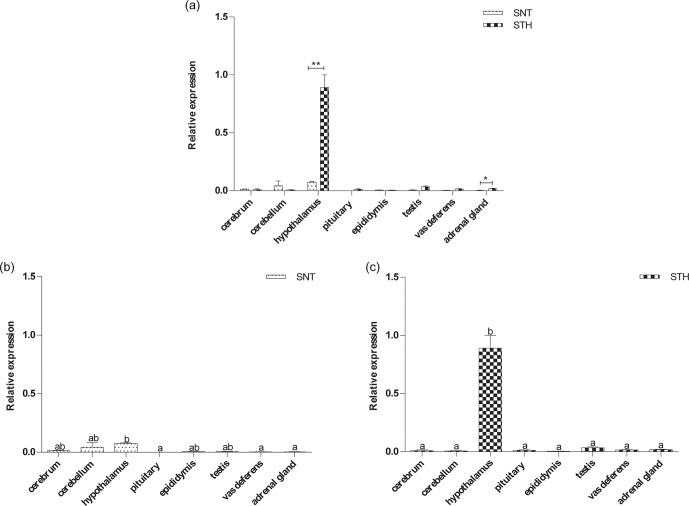
The expression of *KISS1* in eight tissues of small-tail Han sheep (STH) and Sunite sheep (SNT): **(a)** the comparison of the expression between SNT and STH, **(b)** the expression in SNT and **(c)** the expression in STH. Means with different superscripts are significantly different (P<0.05). The significant results with a p value lower than 0.05 are given one asterisk (${}^{*}$) and lower than 0.01 are given two asterisks (${}^{**}$), respectively.

The results are shown in Fig. 4a. *KISS1* was specifically expressed in hypothalamus, and the expression of *KISS1* in hypothalamus is significantly higher in STH than SNT (P<0.01). The expression level of *KISS1* was significantly higher in hypothalamus than that in pituitary, vas deferens and adrenal gland (P<0.05) in SNT (Fig. 4b); it was significantly higher in hypothalamus than other tissues in STH (P<0.05) (Fig. 4c).

## 4 Discussion

### 4.1 * DIO2* expression in seasonally estrous and year-round estrous sheep

*DIO2* is known to be crucial for the seasonal estrous mode of female animals. It plays an important role in thyroid hormone metabolism and regulation (Park et al., 2018). *DIO2* converts inactive (thyroxine, T4) to “active” thyroid hormone (3,5,3${}^{\prime }$-triiodothyronine, T3) and plays a significant role as a determinant of the final concentration of T3 (Dunn et al., 2017; Lomet et al., 2018; Dardente et al., 2019). Previous reports had found that *DIO2* was upregulated in breeding compared to nonbreeding testes in male lizards, while *DIO3* was upregulated in breeding ovaries in female lizards (Kang et al., 2020). Trivedi et al. (2019) found that *DIO2* and *EYA3* were highly expressed, and *DIO3* and *GnIH* were lowly expressed in long photoperiods, concomitant with testis recrudescence in male migratory red-headed buntings. Therefore, the *DIO2* gene has a certain impact on male reproduction. To our knowledge, no research on the expression of *DIO2* in rams has ever been reported. In the present study, the highest expression of *DIO2* was detected mainly in pituitary in STH and SNT rams. It further confirmed that *DIO2* is associated with the pituitary function.

Studies had found that *DIO2* is widely expressed in the cerebrum, cerebellum, thyroid and testis in Brandt's vole and found that the core function of the *DIO2* gene should be restricted in response to the photoperiod rather than factors directly regulating gonadal development (Liu et al., 2017). In addition, some researchers had found that *DIO2* was expressed in the testes in rat and found *DIO2* expression in adult rat testis was consistent with the participation of thyroid hormone in testicular function (Romano et al., 2017). In rodents, *DIO2* was highly expressed in the hypothalamus (Tavolaro et al., 2014). In this research, *DIO2* was shown to be highly expressed in the pituitary and widely expressed in the eight tissues. The result is similar to the study on Jining Grey goat and Liaoning cashmere goat, in which it was found that *DIO2* was expressed in multiple tissues such as the cerebrum, cerebellum, hypothalamus, pituitary, ovary and uterus, and it was highly expressed in the pituitary and uterus (Huang et al., 2016). These indicated that its function is diverse and that it plays a critical role in the pituitary for ram seasonal estrus.

The expression of *DIO2* in the hypothalamus, pituitary and testis is higher in STH than in SNT. This observation was different from previous studies comparing seasonally estrous and year-round estrous sheep (An et al., 2019a, b), in which ewes with seasonal estrus were reported to have a higher expression of *DIO2* in the gonad tissues, which implies that rams may have a different regulation mechanism in estrus compared to ewes. Considering the function of *DIO2* in conversion of thyroid hormone activity, it seems plausible that *DIO2* may have a certain positive effect on ram year-round estrus. Of course, further studies should be performed to deeply investigate the relationship between *DIO2* and ram estrus.

### 4.2 *EYA3 *expression in seasonally estrous and year-round estrous sheep

*EYA* genes had been identified as homologues of *Drosophila* eyes absent (*EYA*) genes. The eyes absent family has a protein phosphatase function, and its enzymatic activity is required for regulating genes encoding growth control and signaling molecules, modulating precursor cell proliferation (Li et al., 2003). The highly conserved vertebrate homologues Six1–6 (Oliver et al., 1996), Eya1-4 (Xu et al., 1997) and Dach1-2/Ski/Sno (Hammond et al., 1998) are co-expressed in multiple organs, including eye, inner ear, pituitary gland, muscle and kidney.

*EYA3* can be expressed in thyroid-stimulating cells in the PT region, and it activates or inhibits the hypothalamic–pituitary–gonadal axis through the TSHβ-DIO2-TH pathway to achieve the regulation of seasonal estrus in sheep (Yoshimura, 2013; Dardente et al., 2014). Previous research had revealed that *EYA3* plays an important role in regulating* TSH*β in male CBA/N mice. *EYA3* and its partner *SIX1* synergistically activate *TSH*β expression, and this activation is further enhanced by *TEF* and *HLF* (Masumoto et al., 2010). Likewise, long photoperiods caused a significant increase in *EYA3*, *CGA*, *TSH*β and* DIO2*, followed by changes in testis size and plasma level of testosterone in male red-headed buntings (Park et al., 2018). Thus, *EYA3* is an important candidate gene in male seasonal estrus.

Previous studies had shown that *EYA3* as a photoperiod-induced gene is highly expressed in pituitary in female sheep, mice and birds (Dupré et al., 2010; Mishra et al., 2017; Masumoto et al., 2010). In this study, *EYA3* was expressed in all eight tissues in rams, which implies that it plays a role in promoting the differentiation of many tissues. This is in agreement with *EYA3* being widely expressed in the pituitary, pineal gland, cerebrum, cerebellum, hypothalamus, fallopian tube, ovary, adrenal gland and kidney in seasonal estrous and year-round estrous ewes (Xia et al., 2018). The highest expression of *EYA3* in testis in STH ram indicated that *EYA3* is associated with the testis function.

In addition, we compared the expression level of *EYA3* in the selected eight tissues between two sheep breeds. We found the expression levels of *EYA3* were significantly higher in cerebellum, hypothalamus, pituitary, testis and vas deferens of STH rams compared with SNT rams. Given the expression of* EYA3* gene in the pituitary in ewes (Xu et al., 1997; Dupréet al., 2010), we speculated that its regulatory mechanism was similar in rams. *EYA3* may have a certain positive effect on ram estrus.

### 4.3 * KISS1* and *GPR54 *expression in seasonally estrous and year-round estrous sheep

Besides *DIO2* and *EYA3*, the molecular mechanism of seasonal estrus involves the downstream signaling factor, including *KISS1* and *GPR54*. They can regulate the release of hypothalamic gonadotropin-releasing hormone (GnRH), which in turn affects the secretion of follicle-stimulating hormone (FSH) and luteinizing hormone (LH), and are closely related to the onset of puberty and may be key genes for mammalian sexual maturity (Novaira et al., 2009; Papaoiconomou et al., 2011). *KISS1* and *GPR54* expression in mouse, rat, rhesus monkey and human testes indicates that this system has an autocrine or paracrine effect in the testis (Ohtaki et al., 2001; Terao et al., 2004; Tariq et al., 2013). In addition, they are contributing to stimulate testosterone secretion, testis maturation, gametogenesis and spermatozoid maturation in epididymis through FSH and LH (Scarlet et al., 2017; Feng et al., 2019). Smith et al. (2005a, b) found that *KISS1* mediates testosterone and estrogen feedbacks on GnRH neurons and differentially responds to sex steroids. Exogenous administration of kisspeptins to sexually inactive male Syrian hamsters reverses the inhibitory effect of nonbreeding season by re-activating the HPG axis (Revel et al., 2006). Therefore, *KISS1* and *GPR54* have a certain impact on male seasonal estrus. But no evidence of the effect of *KISS1* and *GPR54* on seasonal estrus in rams has been reported so far. Therefore, *KISS1* and *GPR54* were selected for investigation of the seasonal estrus of rams in this study.

*KISS1* was expressed in cerebrum and placenta in humans (Muir et al., 2001). Further studies revealed that *KISS1* was expressed with high level in the hypothalamus in pigs (Tomikawa et al., 2010). *GPR54* gene was widely expressed in many tissues in pigs, mice and Siberian hamsters (Li et al., 2008; Shahed et al., 2009; Herbison et al., 2010). *KISS1* and *GPR54* have also been implicated in regulating the estrus cycle of seasonal breeders and in the control of lactational amenorrhea (Colledge, 2008). Our results demonstrated that *KISS1* was specifically expressed in hypothalamus both in SNT and STH. This is consistent with previous studies about seasonal estrous and year-round estrous goats (Huang et al., 2015) and ewes (An et al., 2019a). In addition, *GPR54* was widely expressed in all selected tissues and highly expressed in the testis, cerebellum, pituitary and vas deferens, which implied that *GPR54* may be positively correlated with the seasonal estrus of rams.

Numerous studies revealed that *KISS1* and *GPR54* had effects on testis in goats, mice, monkeys and humans (Terao et al., 2004; Tariq et al., 2013; Samir et al., 2015; Mei et al., 2013). In the present study, we compared the expression level of *KISS1* and *GPR54* in the testis, epididymis, and vas deferens between two sheep breeds. We found the expression levels of *GPR54* in the testis and vas deferens of STH are significantly higher than in SNT rams. The *KISS1 *expression had no significant difference in testis between STH and SNT, while it was significantly higher in STH than in SNT in hypothalamus. Considering the core function of the hypothalamus in seasonal estrus, we speculated that *KISS1* may play a key role in year-round estrus in hypothalamus of rams, similar to their reported functions in other mammals. The results of *GPR54* was in agreement with Han et al. (2020), who found *GPR54* was expressed in sertoli cells, leydig cells and spermatids, suggesting the local expression of *GPR54* in goats' testes and its autocrine role in leydig cells. Therefore, we concluded that the year-round estrus of rams might be due to high expression level of the *GPR54* gene. Of course, further studies are needed to investigate the relationship between *KISS1* and *GPR54* and ram estrus in more depth.

## 5 Conclusions

This study describes the expression pattern of four HPG axis-related genes in year-round estrous (STH) and seasonal estrous (SNT) rams. We found that *DIO2* was highly expressed in pituitary, and *KISS1 *was specifically highly expressed in hypothalamus. The expression levels of *EYA3* and *GPR54* were high in hypothalamus, pituitary and testis, and the expression levels of the four genes are all higher in STH than SNT. The results suggested that the four genes may regulate the estrous mode of ram via HPG axis, and they may play positive functions in the ram year-round estrus. However, the specific mechanism remains to be further explored. This is the first systematic analysis of tissue expression patterns of the HPG axis-related genes in rams.

## Data Availability

The data sets are available upon request from the corresponding author.
